# Conserved intron positions in ancient protein modules

**DOI:** 10.1186/1745-6150-2-7

**Published:** 2007-02-08

**Authors:** Albert DG de Roos

**Affiliations:** 1 Syncyte BioIntelligence, P.O. Box 600, 1000 AP, Amsterdam, The Netherlands

## Abstract

**Background:**

The timing of the origin of introns is of crucial importance for an understanding of early genome architecture. The Exon theory of genes proposed a role for introns in the formation of multi-exon proteins by exon shuffling and predicts the presence of conserved splice sites in ancient genes. In this study, large-scale analysis of potential conserved splice sites was performed using an intron-exon database (ExInt) derived from GenBank.

**Results:**

A set of conserved intron positions was found by matching identical splice sites sequences from distantly-related eukaryotic kingdoms. Most amino acid sequences with conserved introns were homologous to consensus sequences of functional domains from conserved proteins including kinases, phosphatases, small GTPases, transporters and matrix proteins. These included ancient proteins that originated before the eukaryote-prokaryote split, for instance the catalytic domain of protein phosphatase 2A where a total of eleven conserved introns were found. Using an experimental setup in which the relation between a splice site and the ancientness of its surrounding sequence could be studied, it was found that the presence of an intron was positively correlated to the ancientness of its surrounding sequence. Intron phase conservation was linked to the conservation of the gene sequence and not to the splice site sequence itself. However, no apparent differences in phase distribution were found between introns in conserved versus non-conserved sequences.

**Conclusion:**

The data confirm an origin of introns deep in the eukaryotic branch and is in concordance with the presence of introns in the first functional protein modules in an 'Exon theory of genes' scenario. A model is proposed in which shuffling of primordial short exonic sequences led to the formation of the first functional protein modules, in line with hypotheses that see the formation of introns integral to the origins of genome evolution.

**Reviewers:**

This article was reviewed by Scott Roy (nominated by Anthony Poole), Sandro de Souza (nominated by Manyuan Long), and Gáspár Jékely.

## Background

The question about the origin of introns is fundamental for an understanding of the evolution of the genome. Historically, there have been two opposite camps that try to explain the origin of introns (see [1-4]). The 'introns early' school stated that introns arose in ancient genes and were subsequently lost in prokaryotes [5,6], while the 'introns late theory' maintained that the spliceosomal introns were inserted in the eukaryotic lineage into primordial continuous protein-coding regions [2,7-11]. The introns-early theory has been modified to include the insertion of introns later in evolution, while maintaining the role of early introns in creating protein diversity [12-14]. Proponents of both introns-early and introns-late now agree that spliceosomal introns and the spliceosome already existed in the most recent common ancestor of living eukaryotes [15]. Thus, the debate of the timing of the origin of introns has shifted to an origin before or after the prokaryote-eukaryote split. However, even in an introns early scenario, the question remains if and how introns were inserted into genes, since the origin of introns is intricately connected to the evolution of the spliceosome. Also, introns-first hypotheses have been proposed that do not assume intron insertion, but trace introns to the very early origins of the genome [16-18], and position the origin of the spliceosome directly to the first generation of multiexon genes.

In all introns-early scenarios, one would expect the presence of conserved introns between orthologous proteins that diverged before the eukaryotic lineage. There have been studies that used phylogenetic comparisons in an attempt to show that intron position is conserved and therefore ancient (for review see [2,15,19]). Many introns were found in homologous positions in genes duplicated before the separation of bacteria and eukaryotes [20-23]. Also, it has been estimated that up to one-third [21-23] of modern-day introns are shared between at least two major eukaryotic kingdoms. Although the frequency of gain of introns during evolution is still a matter of debate [24-26], it is probable that extensive loss of introns has occurred in the course of evolution. Presently, consensus seems to be that at least some introns were present in the last common eukaryotic ancestor and others were gained later in evolution (for recent reviews see [27,28]). However, the exact timing of the origin of introns before or after the eukaryote-prokaryote split remains a matter of debate.

The formation of multimodular proteins by exon shuffling [6,29] has been proposed to be a possible functional role for ancient (i.e. present before the eukaryote-prokaryote split) introns. The 'Exon theory of genes' proposes that ancient introns would have aided in the creation of early protein diversity by providing the actual sites of recombination in the process of exon shuffling [30-32], and could have aided in creating the first functional protein modules. The functional role of introns may not be limited to shuffling entire domains, but may also have been responsible for the generation of the modules themselves. Recently, a similar scenario has been proposed in a mechanistic reconstruction of the origin of splicing which placed the evolution of the spliceosome at the origin of exon concatenation and shuffling [18]. Other introns-first hypotheses have been proposed in which intronic catalytic RNA introns predated exons [17] or where introns were formed in the early recombination process that involved splicing [16]. However, it has proven difficult to find supporting experimental evidence for an ancient origin of introns.

Support for a functional role of introns in exon shuffling has come from the distribution of intron phase and the excess of symmetrical internal exons, [12,33] proposed to facilitate shuffling between same-phase introns. Also, intron-exon boundaries show a correlation with three-dimensional structure of protein modules [12,34-36]. However, it is still uncertain whether or not ancient eukaryotic multi-domain protein tend to contain introns between the domains (see [28]). A further substantiation of the occurrence of introns in proteins that arose before the prokaryote-eukaryote split and the possible role of introns in exon shuffling or protein domain formation is therefore warranted. In this study, the hypothesis that introns originated in ancient (before the prokaryote-eukaryote split) genes and played a role in exon shuffling or protein domain formation, was further explored.

A large-scale search was performed that identified conserved introns and which allowed the gene sequence that harboured these introns to be studied. It was also found that the presence of an intron is positively correlated to the ancientness of its surrounding exon sequence, suggesting a role for ancient exons as the building blocks of the early protein modules.

## Results

### A set of potential ancient splice sites

**Figure 1:**
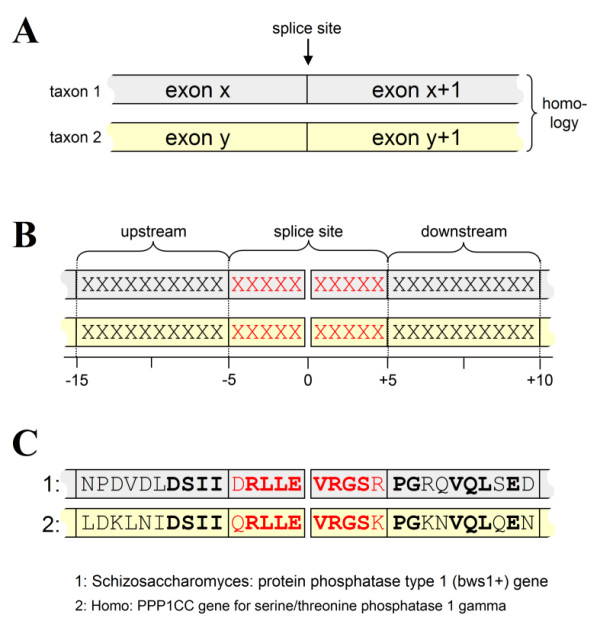
Experimental set-up to select putative conserved genes from an exon/intron database. **A**. Conserved introns were defined as i) homologous exon gene sequences that ii) shared an intron position and iii) were from different taxa that split near the root of the Eukaryotic Tree. The actual splice site sequence was derived from concatenating subsequent exons (exon x and exon x+1, where x is exon number) using SQL and performing a full database scan. **B**. A sequence of 10 residues around the intron position was taken (5 pre-intron and 5 post-intron) as the splice site (in red), while a further homology was determined up- and downstream of the intron (each 10 residues; in black). For a conserved intron, at least 6/10 splice site residues should match and a further 6 matching residues in the 20 residues up- and downstream. **C**. Example of a conserved splice site (in red) with a 8/10 splice site match (in bold) that shows more sequence homology further away from the splice site (black) since 10/20 residues (in bold) match. The sequences shown are from a protein phosphatase that is conserved between animal (human) and fungi (Schizosaccharomyces).

An experimental set-up was defined that allowed for a systematic search for potential conserved splice sites in a genomic intron-exon database derived from GenBank (ExInt; [37]). A conserved splice site was defined as an amino acid sequence that has the same intron position and splice site sequence in two distantly-related protein sequences (Fig. 1A). With this bioinformatics approach, each potential match will be found, no matter how many other non-conserved introns are in the database. To ensure that selected proteins diverged close to the root of the eukaryotic tree, only matches were allowed between two of the following kingdoms: Protists, Fungi, Animals and Plants. This setup was translated into a database SQL query where a reconstructed 10-residue splice site was compared to all other splice junctions in the database (Fig. 1B). Sequences that shared an intron at the same amino acid position from different eukaryotic kingdoms with at least 6 identical residues from the 10-amino acid splice site region were selected. It was found that the combination of a 6 out of 10 identical residues around a common splice site selected mostly for sequences that also showed sequence similarity further removed from the splice sites (Fig. 1C), indicating that these sequences were homologous. Sequences that did not show at least 6 more identical residues from the 20 residues that were located further away from the intron were discarded as possible false-positives due to a chance match on splice site similarity. This resulted in a set of putative conserved splice sites between (co-)orthologous protein domains.

**Figure 2:**
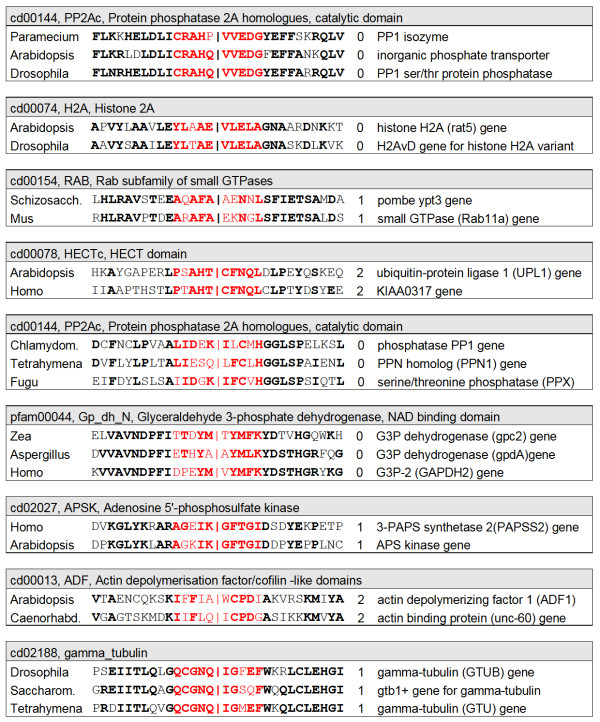
Examples of putative conserved introns. Sequences were selected that had splice site regions (color red) in which at least 6 out of 10 residues were similar (in bold). Of those, only sequences were selected where also at least 6 residues up- and downstream of the splice site matched (in black, matching residues in bold). This minimized false-positive conserved splice sites, although most sequences that had a similar splice site (> 5/10 similar residues) already showed homology in the rest of the sequences. All matching sequences are between distantly related eukaryotic kingdoms, e.g. between Protists, Fungi, Animals and Plants, ensuring that a match would have originated close to the root of the Eukaryotic Tree. For each set, its conserved domain as retrieved from CDD (shaded box) is shown, while the columns show the species name, the sequence around the splice site (splice site given by | symbol), the phase of the intron and a shortened gene annotation.

Based on the method shown above, homologous sequences were identified with an identical intron position relative to the amino acids sequence. Figure 2 shows some examples of the conserved splice sites that were found. The entire set of conserved genes with a shared intron position set used comprised 459 putative conserved introns [see additional file 1] shared between eukaryotic kingdoms (Plants, Animals, Fungi, and Protists). Strongly homologous sequences from the same kingdom combinations where removed (e.g. the gpc2 gene of Zea and the GapC gene of Arabidopsis both share an intron with the Human GAPDH2 gene but its sequences are very similar), further reducing the set to 251 putative conserved introns (see additional file 'deroos-conserved sets.zip'). The intron phase was identical in almost all of the matching sequences found (218/251 = 87%), showing that these sequences shared the exact ribonucleotide splice site relative to the amino acid sequence. This indicates the non-random occurrence of introns position in the selected genes, as expected for conserved introns. Most (83%) of conserved splice site sequences could be found in the conserved domain database at NCBI. Some sequences with conserved introns were represented by three eukaryotic kingdoms, and these included phosphatases, kinases, small GTPases (Rab, Rho), ubiquitin, triosephosphate isomerase, alcohol dehydrogenase and actin. Most of these genes can be considered to have existed before the eukaryote-prokaryote split, showing that the selection method can positively identify conserved splice sites in ancient genes. Thus, based on the broad phylogenetic distribution of the set of splice sites and the conserved intron phase, the selected sequences can be considered to be potential conserved splice sites.

### Shared introns in ancient protein domains

**Figure 3:**
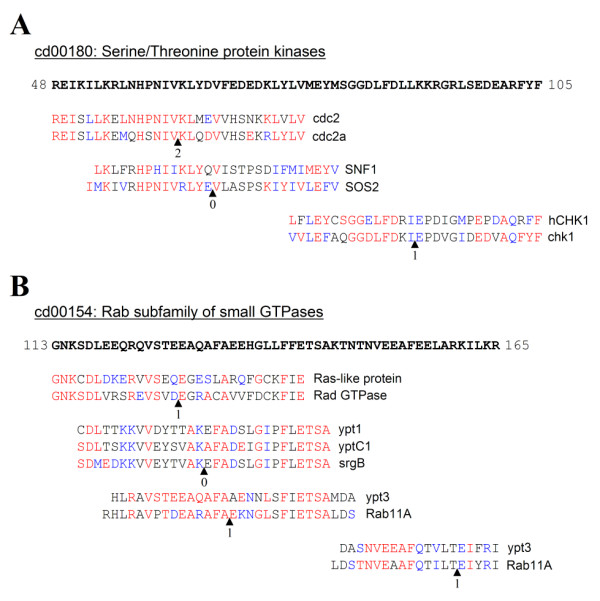
Conserved splice sites map to conserved protein domain sequences. Parts of consensus sequences of conserved domains from serine threonine kinases (**A**; CDD# cd00180) and small GTPases (**B**; CDD# cd00180) from the CDD database are shown in bold on top, while short sequences from different eukaryotic kingdoms containing a conserved splice site are aligned with this consensus sequences. Identical amino acid residues to consensus sequences are shown in red, homologous amino acid residues in blue and no amino acid homology in black. Colour scheme is identical to the one used by the CDD search at NCBI. Arrow heads indicate (conserved) intron position and phase. To the right of the sequence, the abbreviation of the gene annotation is shown.

**Figure 4:**
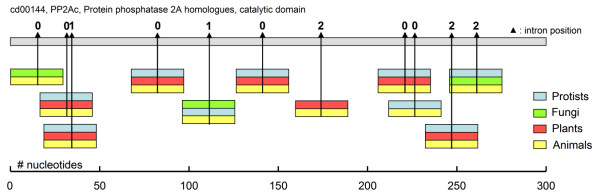
Conserved splice sites mapped to the conserved domain of an ancient phosphatase. Eleven sequences with conserved splice sites could be mapped to the consensus sequence of ancient phosphatase (CDD identifier cd00144), cf. Fig. 3. The 300 amino acid-long conserved protein domain is shown on the top, with a residue number indicated at the bottom. This sequence can be found by using the entry cd00144 in the CDD at NCBI. The short colored boxes represent a 30 amino acid long splice site region, two examples of a 'cd00144-related' sequence can be found in Fig. 2. Conserved splice sites positions are indicated by the vertical lines on the conserved domain sequence and arrowheads, while intron phase is also shown.

The names of the genes and the observed conservation of the sequences already suggested a common function of the sequences involved (cf. Fig. 2), as expected for conserved introns. Searching the conserved domain database (CDD) at NCBI revealed that these sequences with putative conserved introns could almost all be matched to the consensus sequence of conserved domains. The CDD includes a consensus sequence of the domains and these were used to map the different splice site sequences to these domains. Multiple conserved introns were found in some conserved protein domains and conserved introns were sometimes represented in three different taxa. Three conserved splice sites were found in a 30-residue long part of the serine/threonine kinase domain, while four conserved splice sites were found in the Rab-domain. Figure 3 shows the mapping of some of the conserved splice sites to the consensus sequence of the conserved domains of serine/threonine kinase (CDD entry number cd00180; **A**) and Rab related genes (cd00154; **B**). The largest number of conserved splice sites was found in the consensus sequence of the catalytic domain of protein phosphatases 2, comprising a large family of serine/threonine phosphatases (Fig. 4). In this ancient phosphatase domain of 300 nucleotides, 11 conserved splice sites were found, each with an identical intron phase (position relative to codon). The smallest exon size that was found in this study comprised 4 nucleotides, a 2 amino acid-long exon flanked by a phase 0 and a phase 1 exon (see Fig. 4; between 2^nd ^and 3^rd ^intron). Members of the Rab related genes are common throughout the eukaryotic tree, and members of the serine/threonine kinases and the protein phosphatase 2 domain are also present in Bacteria and Archaea, indicating their ancient origin. These results show that the mapping of these individual conserved splice sites to its ancestor protein can reveal the wide-spread occurrence of these sites in ancient proteins.

### Quantifying the relation between intron and sequence conservation

From an introns-early perspective, the conserved introns that were found in ancient protein domains, such as those for phosphatase and kinase, could represent the remnants of the introns between the exons that could have been used for the exon concatenation or shuffling in early genome evolution. However, since the query used to look for ancient introns is based upon selecting conserved sequences, the introns found in ancient protein modules could also represent a sample of a larger number of randomly distributed introns and not be specific for ancient genes. This can be investigated by studying the relation between the ancientness of the selected sequences and the presence of an intron where a positive correlation would be expected according to the Exon theory. No relation could point to a bias in the results, where the putative ancient introns are just a sub-selection of conserved sequences that happen to contain a conserved intron.

**Figure 5:**
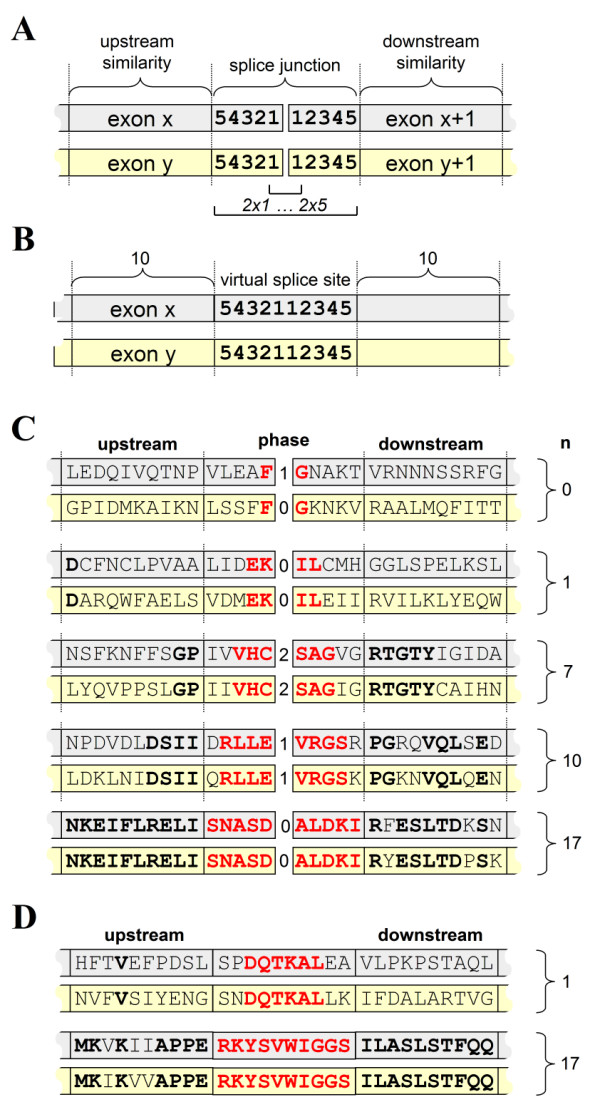
Experimental set-up to determine the effect of intron presence on the ancientness of the sequence it is in. **A**. The number of identical splice site residues was varied between 2 (one residue on both sides of the intron position) to 10 (2*5). The maximum number of 10 represents then a perfect match of 10 residues around a splice site. As an indication of sequence ancientness, the number of matching residues further up- and downstream was calculated. The maximum number that can be reached is 20 (10 on each side) representing a perfect match in this region. **B**. Control situation that described the setup with identical parameters but without an intron. All sequence comparisons in this control were thus performed relative to a virtual splice site (intron position). **C**. Example sequences that have respectively 1, 2, 3, 4 and 5 fixed identical sequences at each end of their splice site (in red). The fixed identical sequences around the splice site are shown in bold red. Identical residues further up- and downstream of the splice site that are used to determine general homology of the sequences are shown in bold black characters. **D**. Similar results as in C, using sequences without a splice site. Shown are sequences with 6 and 10 identical residues around the virtual splice site.

In order to quantify the relation between ancientness of the gene and the presence of an intron, an experimental setup was used in which the effect of the splice site on the ancientness of the sequence could be studied. A variable-length splice site region was defined in which the length of the identical exon sequence could be stepwise increased from 2 to 10 identical residues by adjusting the SQL query (Fig. 5A). A measure of the ancientness of the surrounding sequence was taken by calculation of the number of identical sequences up- and downstream of the splice site. In order to show the specific effect of the intron, a control situation was created that had the same selection criteria, except for the presence of an intron (Fig. 5B). It was found that when short identical splice site sequences (1 to 2 residues on each side) were taken, the sequences did generally not show any further similarity, due the relatively high random occurrence of these short splice site sequences. Longer identical sequence adjacent to the splice site (4 to 5 on each side) selected mostly conserved genes, as measured by the number of identical residues up- and downstream of the splice site (Fig. 5C). Also in the absence of introns, selection for a short region of similarity yields in general non-conserved sequences, whereas a match of a stretch of 10 identical amino acids between distantly-related kingdoms almost always means that there is more homology in the sequence concerned (Fig. 5D). In general, these results show a relation between the ancientness of the genes and form the basis for an analysis of the effect of an intron.

### Positive correlation between intron presence and ancientness of sequence

**Figure 6:**
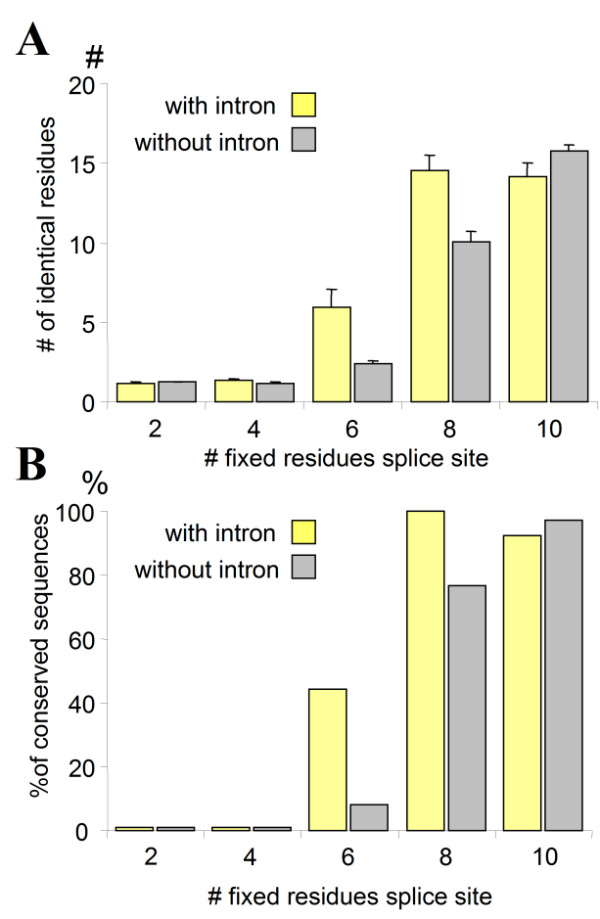
Intron presence and ancientness of gene sequence. **A**. Average number of identical residues in the homology regions between sequence pairs as a function of the length of identical residues (x) around the (virtual) splice site. Sequences with a splice site were compared with control sequences without a splice site (cf. Fig. 5A and B). Maximum number of identical residues is 20, representing 100% similarity in this region. Error bars represent standard deviation with N = 650 (x = 1), N = 564 (x = 2), N = 34 (x = 3), N = 11 (x = 4) and N = 27 (x = 5), and for the control N = 267, 695, 199, 60 and 65, respectively. **B**. The same data set as in A, but now the percentage of *conserved sequences *as a function of number of fixed identical residues is shown. Conserved sequences were defined as having 6 or more identical residues (apart from the identical residues in the splice site; red in Fig. 5) in the two regions of homology (black in Fig. 5), dividing the data in a non-conserved and a conserved gene set. Cf. Fig. 5C where the first 2 sequences would be considered non-conserved, and the last 3 would be conserved.

Using the described set-up above (Fig. 5), the length of the splice site was varied and the effect on the ancientness of the genes in each set was determined. As expected, short stretches (2–4 identical residues) hardly yielded conserved genes, while with longer (10 residues) regions only conserved sequences were found, both with and without an intron. However, with sequences of intermediate length (6–8 residues), sequences with an intron are generally more conserved than their counterpart without an intron, as measured by the number of identical residues in the region directly outside the defined splice site (Fig. 6A). An analysis of the distribution histograms (not shown) indicated that the increase in conservation was due to a recruitment of conserved genes, and not a gradual increase in general homology. Sequences that had 6 out of 20 additional residues in common (excluding the splice site region) were considered conserved. When instead of the number of shared residues, the sequences were divided in non-conserved (<= 4 residues) and conserved (>= 6) sequences, the effect of the presence of the intron was more pronounced (Fig. 6B). These results show that the presence of intron has an effect on the percentage of ancient sequences obtained, which is not expected when the method just selected ancient genes that happened to share an intron, regardless of antiquity.

A second, independent measure of the ancientness of the sequences that contain conserved introns can be made based on their presence in the main Life groups Bacteria, Archaea and Eukarya. Instead of counting the number of common residues as done in Fig. 6, the 30-residue splice site region was used as an input for a BLAST query at NCBI. The phylogenetic distribution of the specific input sequence was included in the output so that the presence throughout the Tree of Life could be determined. A number of identical amino acids residues of 6 (2 × 3) around a (virtual) splice site was used as the number that could best distinguish between ancientness of the genes (cf. Fig. 6B). From this set, only the splice site sequences were taken that did not show considerable sequence similarity apart from the splice site region itself. In the set that contained an intron, 42.1% (8/19) of these splice site sequences with low similarity except from the splice site, were represented by a protein that was found between at least two of the three main kingdoms. In contrast, in sequences without an intron, only 19.6% (10/51) represented genes found between the three main kingdoms (p-value = 0.053 using T-test). Using the presence of the gene sequence in the conserved domain database as another measure of ancientness, in the set with introns, 52.6% (10/19) was a member of a conserved domain protein family as derived from a CDD search, versus 23.5% (12/51) in the sequences without an intron (p-value < 0.018 using T-test). In both sets, the average number of residues up- and downstream of the splice site was similar, 0.89 ± 0.19 (mean ± sem; n = 19) with intron and 1.13 ± 0.14 (n = 51) with intron, indicating no starting bias towards homologous sequences. These results confirm the basic findings shown in Fig. 6 that introns are positively correlated with the ancientness of its surrounding sequences.

### Intron phase distributions between intron subsets

**Figure 7:**
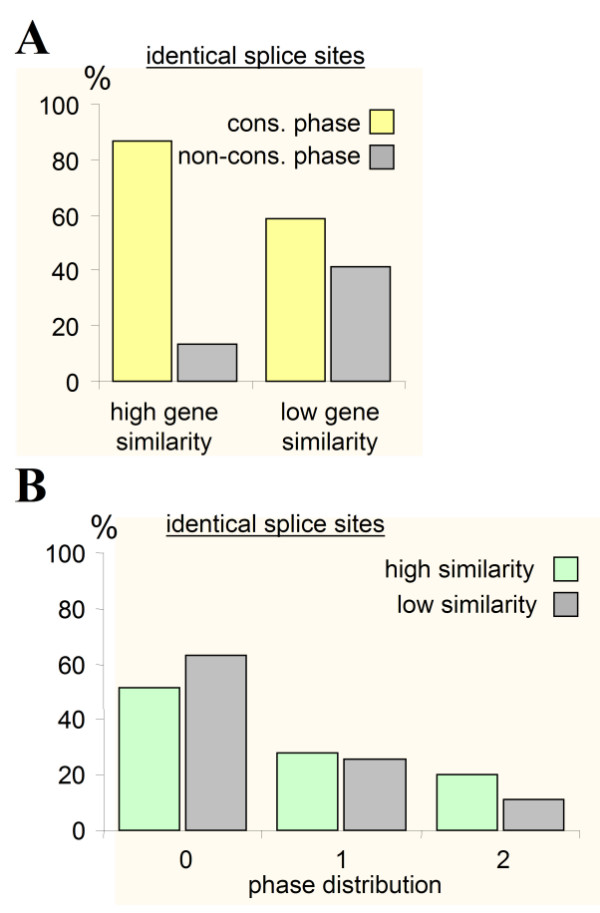
Intron phase and conservation of splice sites. **A**. Percentage of identical intron phases (0:0, 1:1, 2:2), and different phases (0:1, 0:2, 1:2) in a set of paired conserved genes with conserved introns (N = 251) versus a set of non-conserved genes (N = 1231). Both sets had identical splice sites, having 2–10 residues in common (cf. Fig. 5) while only the conserved set also showed homology in the rest of the sequence. Conserved was defined as those sequences that have 6 or more identical residues in the general homology region. **B**. Distribution of intron phase between conserved (N = 217) and non-conserved (N = 723) genes in the sets of identical phases shown in A.

As expected when the introns were present before the protein sequences diverged, intron phase was found to be conserved in the set of putative conserved splice sites (cf. Fig. 2). However, the same pattern could have been observed when introns were inserted into specific target sequences (proto-splice sites). To distinguish between these possibilities, the conservation of intron phase was compared to sets of sequences with matching splice sites, but with no other similarities of the sequence. Figure 7A shows that intron position (phase) is not conserved between introns that are only identical at the splice junction, but was conserved when such a sequence was embedded in a conserved gene. These results show that it is the ancientness of the gene that determines the phase of a specific intron and not the splice site sequence, and argue against parallel insertion of introns in a proto-splice site. The distribution of intron phase itself showed no direct relation to the ancientness of the sequence since the subset of introns that are located in ancient sequences has a similar phase distribution as introns in the general population (Fig. 7B). The conservation of intron phase and distribution are in line with an early establishment of intron phase.

## Discussion

### Methodological aspects in the study of the origins of intron

In this study, a straightforward search method in a relational database was used to identify candidate conserved introns that could have delineated ancient exons. One of the advantages of this method was that the definition of a conserved intron can be easily translated in terms of the query language SQL. Also, the method allowed to precisely define and control the variables used, permitting quantitative analysis of the queries. A strict definition of a conserved splice site was used where not only the position, but also the sequence was conserved and where intron-sliding was not allowed. Other datasets of conserved introns have been published [22,23] that showed a large number of introns that are shared between eukaryotic kingdoms. In these studies, first common eukaryotic genes were selected using a BLAST search, after which the number of conserved intron positions in those genes was determined using an alignment algorithm. Although yielding many more conserved introns than in this study, the complexity of the used algorithms to select a set of genes and align them, makes it difficult to interpret different intron characteristics within the given set of conserved introns.

The stringency of the method used in the present article, a high similarity over a short sequence, was designed to select virtually no false-positive conserved introns. The set of conserved introns is therefore not exhaustive, but was intended to identify conserved introns that would satisfy the definition for ancient introns. Since the method involves only a short sequence for a potential match, it does not rely on any *a priori *homology outside this region, allowing to find conserved splice sites in sequences from genes that show little overall homology, for instance in co-orthologous genes. The strict definition of a conserved splice site therefore limited the result set, but did select a well-defined subset of splice sites that could represent the ancient exon building blocks of protein modules. In summary, the present methodological concept can be seen as complementary to more conventional methods to study the conservation of introns.

### Ancient origin of introns?

The results in this and other studies identified conserved introns that are shared between different eukaryotic kingdoms. The rationale behind this article was based on the assumption that exons originated at the origins of the genome itself (see [18] and represent the building blocks of functional modules (c.f. the Exon theory of genes). In this view, the conserved introns found could represent the last remnants of a slow streamlining process that tends to eliminate useless DNA, or alternatively these introns have acquired a function that would prevent these specific introns from being pruned from the genes. The number of splice sites in some conserved protein domains (e.g. phosphatase in Fig. 4) suggest that ancient protein domains were composed of many exons (or contained many introns), which has also been proposed in other studies [22,36,38]. The current study suggest a minimum exon length around 4 nucleotides, similar to modern minimum reported exon lengths (see [39] for exon sizes). Also, the present study suggests that only a fraction of the ancient intron positions remained in the modern genes, in line with current models that assume massive intron loss (for recent reviews see [19,26-28]. No relation between the ancientness of the sequence and the absolute value of the phase was found, indicating that the current phase distribution is similar to the ancient one. This would also be in line with an ancient origin of introns where phase was already determined and mainly intron loss occurred. This is in contrast with other studies that have showed correlations with phase arguing for an introns-early [36,40], or introns-late scenarios [41].

In an introns-early/Exon theory scenario, one would expect that conserved introns would be preferentially found in sequences that have been conserved. In this article, a positive correlation was shown between intron presence and ancientness of the sequence it is in. Although providing circumstantial evidence for an introns-early scenario, it will be difficult to discriminate between an ancient presence and loss in prokaryotes (cf. introns-early) versus an insertion early in the eukaryotic tree (cf. introns-late). However, the emerging view of an ancestor eukaryote that already contained a complex, intron-rich genome [[42-44], this study] makes an introns-late scenario mechanistically difficult. In an introns-late scenario, the preferred occurrence in conserved sequences would suggest the insertion of introns very early in the eukaryotic tree, or assume a preferential insertion in ancient genes. It is difficult to imagine how a massive invasion of introns into existing, and essential protein modules early in eukaryotic evolution is feasible without major effects on fitness. Therefore, results that focus on the more mechanistic steps of intron origin may still feed the debate about the origin of introns.

### Exons as building blocks of the genome

In the Exon theory of genes [6,45], it was proposed that introns would specifically delineate functional boundaries, e.g. protein modules, in ancient genes where introns would facilitate the creation of multifunction genes by providing the actual sites of recombination. Recently, it was reasoned based on the engineering paradigm design-by-contract, that exon concatenation of modular exonic sequences was the basis of eukaryotic genome formation [18], providing a mechanistic basis for the Exon theory of genes. In the current study, conserved introns positions were found *within *a variety of ancient protein modules, suggesting that the initial function of exons did not represent the boundaries of functional protein modules, but that the domains itself were assembled from exons. In such a model, individual exons do not have to coincide with functional modules but could represent structural modules or just connectors (like LEGO blocks) with no further intrinsic function, in line with the relative small size of some of the proposed ancient exons. Shuffling and recombination of these building blocks could then quickly generate a large variety of potential functional modules, on which natural selection can then act. It is the author's opinion that such active recombination mechanisms are a requirement for building a genome and may underlie an intrinsic evolvability in the genome. Also, such a scenario fits in a model in which a eukaryotic-like genome was the ancestor genome and prokaryotes were derived from this ancestor (see [46,47]).

## Methods

Large-scale analysis of intron position and exon amino acid sequence was performed using a bioinformatics approach on publicly available genomic databases. The exon/intron database ExInt [37] was used as the main source for the genomic data, while the online conserved domain database (CDD) database at NCBI was used to couple gene sequence to functional domain. The ExInt database of genes from GenBank contains exon and intron attributes such as sequence and phase in a relational database. This database contains three tables, a main table with protein data such as total sequence and gene annotation, an exon table that contained the individual exons, including exon number and sequence, and an intron table that was used to obtain the intron phase. Both intron and exon table were linked through the main table by a unique gene entry identifier. Splice site junctions were determined by joining the end sequence of the first exon with the beginning of the next exon using standard SQL functions. Each splice sequence was then compared to all other splice sequences to find a match. This basically represented an auto-join on the exon table and exon table aliases were used to represent each of the potential splice junctions. Matching on amino acid sequence was performed by standard SQL string comparison functions.

The ExInt database contains a total of 120,573 gene sequence entries with 518,168 exons and 417,178 introns. Genomic databases like GenBank contain significant numbers of redundant sequences that have to be dealt with when performing large-scale comparisons. A large part of the redundancy in GenBank comes from non-identified sequences where a gene was identified by an algorithm that detects open reading frames and introns. The pre-selection on the presence of the word 'gene' in the sequence annotation selected only identified genes and greatly reduced redundancy of the result sets, effectively reducing the size of the database from 120,573 to 27,861 entries. The available species annotation of genes was used to link sequences to their taxonomic category. An additional taxon table was created by selecting representative species from the ExInt database and classifying these into the main eukaryotic kingdoms categories Protists, Fungi, Animals and Plants. The NCBI taxonomy browser was used for the classification of these species. The resulting taxon table contained 94 entries with at least 10 entries within each eukaryotic kingdom, covering 60% of the GenBank entries in the ExInt database and leaving 16,741 gene entries. Since there were more than one species per defined kingdom, result sets generally included homologous sequences when multiple species within a single kingdom gave a match to a single gene from another kingdom. In order to reduce redundancy, results were therefore cleaned by removing data with identical eukaryotic kingdom combinations (e.g. human vs. yeast and mouse vs. yeast was reduced to one entry).

The conserved domain database (CDD) at NCBI [48]was used to identify the conserved domains present in a protein sequence. The short sequence of 30 amino acid residues around the (virtual) splice sites that were retrieved, were used directly to query the CDD for evolutionary conservation using the interactive query functionality of the CDD browser at NCBI. A positive match returned the consensus sequence of the conserved domain and other genes that belong to the same gene family. The CDD default E-value (expectation value) threshold of 0.01 was used in all experiments. Using the interactive information provided by NCBI for each representative species, its distribution throughout the Tree of Life was determined.

All experiments were performed on a normal laptop PC (2 GHz, 512 MB RAM) with Windows XP running a MySQL relational database (version 4). Querying was done using MySQL Control Center. The result of each query was visually inspected for redundancy and specificity and was further manually checked for consistency by using the found sequences to search the PubMed protein sequence. In general, result sets were imported in a new single-table database for further analysis. Analyses for graphical representations and statistics were done in Excel.

## Competing interests

The author(s) declare that they have no competing interests.

## Authors' contributions

AdR designed all the experiments, analyzed the data and wrote the manuscript.

## Reviewers' comments

### Reviewer's report 1

Scott Roy, Allan Wilson Centre for Molecular Ecology and Evolution, Massey University, Palmerston North, New Zealand. Nominated by Anthony Poole.

"Conserved intron positions in ancient protein modules" by de Roos. The paper takes an ingenious approach to the attempt to distinguish between the introns-late and introns-early perspectives. Much previous evidence for the introns-early theory has relied on the relationship of intron positions to the coding frame of the flanking exonic sequence, or to the three-dimensional structure of the corresponding protein, findings whose existence and relevance have not always been accepted by proponents of introns-late. Avoiding these mine strewn landscapes, de Roos investigates another prediction of the presence of introns in LUCA – if introns were (primarily) created in ancient times, they should preferentially fall within ancient sequences. However, I am very concerned about various methodological issues, and therefore suspect that the results do not inform the debate.

#### Author's response

*In concordance with the comments of other reviewers, I have focused less on a discrimination between introns-late and introns-early, but more towards support of the Exon theory of genes. I also discussed the potential bias in the results and the way this was investigated*.

The author presents three related lines of evidence for pre-LUCA introns. First, conserved introns tend to be found in conserved protein domains. Second, among conserved sequences, those that contain introns are more conserved (i.e. sequences conservation extends further along the protein sequence). Third, conserved introns are preferentially found in genes common to all three domains of life.

An alternative explanation for the first finding, that introns tend to be found in conserved protein domains, is that there is a bias in the finding of 'conserved' introns. Here, conserved introns means those introns that share positions across eukaryotic groups in very highly conserved sequences (for instance 6 residues exactly conserved). Similarly, domains are currently generally defined by sequence conservation across long evolutionary distances. Thus, this finding reduces to: introns in short highly conserved sequences are preferentially found in longer highly conserved sequences. This is not particularly surprising. Without a subtle negative control, it is hard to discern a signal of ancientness above this potential bias.

#### Author's response

*I am aware that the finding of conserved introns in conserved domains does not prove an ancient origin, since the search method could not discriminate directly between an insertion early in the eukaryotic tree. However, the Exon theory of genes expects conserved introns in ancient modules and the first part of the article set out to find those potential conserved introns. In this way it is a confirmation of the hypothesis, although it cannot be considered evidence. I designed the experiments in *Figs 5* and *6* to address this point (see below). The nature of the bias and how this has been investigated is now stated explicitly*.

The second finding is curious, but I fear may also be explained by limitations in the methodology. Here, the central finding is that short highly-conserved sequences containing conserved introns are more likely to lie in long highly-conserved sequences than are those that do not contain introns. Given the enormity of the sequence database, it is likely that some highly-conserved sixmers will be false positives – i.e. they will not reflect true homology. The probability of a false positive is greatly reduced by the presence of a conserved intron (the vast majority conserved-sequence, conserved-intron sequences are likely homologous). Thus, the conserved intron-containing set is likely to contain far fewer false-positives than the control set. The higher false positive rate among the control set predicts a lower extent of sequence conservation, just as seen. Thus it is again difficult to discern a signal of ancientness.

#### Author's response

*In order to investigate a potential bias, the experiments in *Fig. 5* were designed, were a control situation was created that used exactly the same methodology, but now without an intron. It was shown that sequences without an intron are less often found within a conserved domain or sequence. In other words, if a sample is taken from a genomic database that selects short, highly similar sequences, the gene it is in is more ancient if an intron is also shared between these sequences. This would not be expected when the sequences with an intron are just a subset of a larger set with conserved sequences regardless of an intron*.

*The assumption that the probability of a false positive is greatly reduced by the presence of a conserved intron was actually tested in the experiments. Although not evidence for the ancient origins, this does strengthen the finding that introns at similar positions in conserved genes are homologous and would be in line with an Exon theory of genes*.

The final finding, that intron-containing matching sequences are preferentially found across domains of life, is not statistically significant: the fraction of intron-containing sequences in 'ancient' genes (8/19) is not statistically different from the fraction of intronless sequences (10/51; *P *= 0.07 by a Fisher Exact Test). Thus while the difference in fractions of ancient genes is suggestive, we cannot currently conclude anything from this finding. In addition, I am concerned that this test may also suffer from problems similar to those discussed above.

#### Author's response

*These sets consisted of sequences where only the splice site sequence matched (2 × 3 residues), but did not show any further substantial gene similarity. It is true that this value is not statistically different, and this is now shown (I found p = 0.053 using Student T test). However, there were more than one test that suggested the ancientness: a) overall sequence similarity, b) representatives of ancient protein modules, and c) representatives of protein modules that are shared between prokaryotes as well as eukaryotes. Although these tests cannot be considered truly independent, the set of controls does suggest that the introns are preferentially located in ancient sequences*.

In addition, the unorthodox methods used (complete sequence conservation over short sequence fragments) are hard to evaluate given the lack of a history for such methods. It is not clear why these methods have been chosen over more traditional tools, which are abundant. This makes it difficult to interpret the data.

#### Author's response

*I have included a paragraph that discusses the method used, and added the result set of potential conserved introns*.

*Assuming an 'Exon theory of genes'-scenario (de Roos, 2005), I set out to look for conserved introns. I defined a potential conserved intron just by a shared intron in a short sequence, without any preselection of the type of genes. Since SQL is a powerful tool to extract specific information from large relational data sources, this quickly led to introns that could be considered conserved. Since sequence matching was done using a simple algorithm that compared sequence similarity, it was easy to control the variables and do the experiments with control sequences as in *Fig. 5. *I believe this is one of the main advantages: the straightforward way of querying makes it possible to design predictive experiments by changing one parameter at a time and use a control situation*.

One further consideration is the usage of the word 'ancient.' This word is traditionally reserved in the context of the intron debate for the period before the divergence of eukaryotes from prokaryotes, and this distinction has become all the more important with the mounting evidence for significant intron presence in early eukaryotes. One way to distinguish the two epochs is between 'ancient' and 'ancient/early eukaryotic'.

#### Author's response

*This is an important distinction and has been made more clear in the article. Although it is difficult to make a distinction between ancient and ancient/early eukaryotic with the experiments presented, the data is in line with ancient origin (before pro/eukaryotic split). I assume, however, that eukaryotes were not derived from prokaryotes, but that they both share an ancestor whose genome was eukaryotic-like*.

Following a long line of previous results, de Roos identifies intron positions that are shared between broad eukaryotic groups. Previous statistical analyses of the numbers and distributions of these patterns have concluded that a substantial majority of these represent ancestral positions, indicating that there were already substantial intron numbers by the time of divergence. While the eukaryotic groups used here (and elsewhere) may not allow for inferring an intron's presence in the last ancestor of all extant eukaryotes, we can now be confident that there were already a very large number of introns in the last ancestor of eukaryotes, since the spliceosome is broadly shared among eukaryotes, and large numbers of intron positions are shared between potentially early diverging eukaryotes and others (Slamovits and Keeling this year solidified the last link, showing conservation of more than 0.5 introns per gene between an excavate species and 'later-diverging' species, confirming Archibald, O'Kelly and Doolittle's 2002 work).

I bring up this in order to point out two things. First, the debate over the timing of introns is now over whether introns arose in a universal ancestor or between the eukaryotic-archaeal ancestor and the last eukaryotic ancestor. Although findings of conservation of introns or splicing-associated features across eukaryotes underscores the fact that introns are very old within eukaryotes, it does not help us to distinguish the timing of origin of spliceosomal introns. Second, it is important to point out that the findings of large number of introns in early eukaryotes are not fundamentally transformative for the introns early/late debate. Though substantial intron presence is clearly resonant with introns-early (and perhaps nearly necessary for the model), it does not contradict the introns-late model. The fundamental tenet of introns-late is that introns arose after the last common universal ancestor, thus intron presence in early eukaryotes only refines the timing of the origin on that model.

#### Author's response

*I rewrote sentences that implied a contradiction of introns-late. Instead I focused on results that are in line with an Exon theory of genes. On the other hand, the patterns of conserved introns in ancient protein modules, phase distributions and correlation with ancientness of genes as seen in this article, may also make a mechanistic model for introns-late more difficult and is therefore relevant to the debate*.

*I think that the most important question in unravelling the origins of introns is whether introns were inserted into preformed genes, or were at the basis of the genes themselves. Insertion of introns at the DNA level seems extremely difficult given the fact that an intron should be excised at the RNA level perfectly in order to be functional. I have not seen a mechanism for gradual spliceosome evolution and intron insertion that could address the negative fitness effects of such a scenario. Moreover, intron insertion models should explain how complex genes arose in the first place without the help of exons. So, as long as the mechanistic puzzles have not been solved, I consider the debate still open*.

In light of this, we can ask whether tests such as those undertaken here are likely to shed light on the introns early/late debate. The presence of shared introns in shared eukaryotic genes does not shed light on whether these introns date to earlier epochs. One possible approach would be to compare intron densities between ancient (shared eukaryotic-prokaryotic) gene and ancient eukaryotic genes. However, even in this case a difference would not be conclusive support for ancient introns, since the origin of many ancestral eukaryotic genes may postdate the origin of introns. And this is leaving aside the differential rates of intron loss across genes, the fact that genes that arise by gene duplication (and then diverge beyond recognition) may retain ancestral introns, and difficulties with distinguishing truly eukaryotic-specific genes from the inability of programs like BLAST to detect homology.

#### Author's response

*Another approach may be to first unravel the mechanistic steps in an Exon theory of genes for building a genome, and then compare the construction of eukaryotic genes with those of prokaryotes and see if they are assembled in a similar way. In this respect, the proto splice-sites as proposed by Dibb and Newman*[49]*could as well represent the remnants of the ancient exon concatenations (see*[18]).

### Reviewer's report 2

Sandro de Souza, Ludwig Institute for Cancer Research, Sao Paulo, Brazil. Nominated by Manyuan Long

Albert de Roos developed a new way to identify conserved intron positions between taxa from different kingdoms of life. Instead of looking for conserved intron positions in know related genes, de Roos searches for conserved "splice sites" in an intron database. By "splice site", he means a segment of protein sequence (10 aa residues) flanking an intron position. He considered an intron position conserved when this "splice site" was conserved (6 out of 10 residues in that window) between two distantly related protein sequences. The strategy may be interesting since it can identify cases missed by other approaches. Although I recognize the efforts of the author in trying to look at this problem with a different, creative strategy, I found the manuscript hard to follow and highly speculative with many assumptions not supported by the data. Furthermore, since this is a new strategy, the author should give more details about the methodology as well as provide more analyses for his dataset. I will list some issues below in a point-by-point basis to make it easier for the author to reply.

#### Author's response

*I have changed the article to focus more on the data that support a 'very early' origin of introns, but does not prove introns-early, and can by itself not disprove introns-late. The article was too strong in that aspect*.

Overall, I have many problems with the "Introduction" section, in which many important references were misquoted or ignored.

• For instance, the original quotation for the introns-early theory is given to Gilbert's 86 paper on RNA world and to a review from Gilbert and colleagues in Cell (also from 86). Here the papers cited should be the Nature 1978 paper from Ford Doolittle and the "Exon Theory of Genes" paper from Gilbert in 1987, which best represent the early phases of the introns-early theory.

• Furthermore, the synthetic theory of intron evolution, developed by myself, Scott Roy and Wally Gilbert is completely ignored.

• In a particular point, the papers cited as evidence for a correlation between intron positions and module boundaries is certainly outdated. Papers from de Souza et al (1996), de Souza et al (1998) and Fedorov et al (2001) were ignored but best represent the conceptual aspect explored by the author.

#### Author's response

*I changed the references and rewrote the introduction*.

1) In the last paragraph of the "Introduction" section, the author state that the rationale for this manuscript was based in the assumption that "*....one would expect to find conserved introns specifically in ancient proteins*". In the way is presented, this sounds wrong. An intron conserved between vertebrates and invertebrates does not necessarily be located in an ancient gene. I think the author should be more explicit by saying that this conservation has to be deep among the four kingdoms that he later will mention.

#### Author's response

*I rewrote the introduction and the articles focuses on the identification of possible ancient introns and their characteristics*.

2) It would be nice to have a more detailed description of the 251 (or 250?) conserved intron positions. The entire list of positions should be available. It was not clear as well the level of stringency used in the analysis. As far as I understood a simple match involving proteins from, for example, human and Arabidopsis was suffice to flag that as conserved.

#### Author's response

*I included the entire result set of the potentially conserved introns, as well as the larger set with strongly homologous genes that was cleaned to obtain the 251 potential ancient introns. The flag 'shared between major eukaryotic kingdoms' was indeed based on the occurrence in plants as well as animals. In order to further qualify for 'conserved', the splice site sequence and intron position should be conserved, as well as show more homology further away from the splice site. The stringency is also discussed in the article*.

3) Since this is a new method, the author should compare his dataset with other datasets. For example, the dataset reported by Rogozin et al (Curr. Biol 13:1512–1517, 2003) was analyzed by these authors and by Roy and Gilbert (PNAS 102:1986–1991, 2005). The datasets are apparently significant different. They should be since the methodology is different but the author needs to explore these differences. For instance, I am surprised by the low number of conserved intron position identified by de Roos. In the dataset mentioned above, there were almost a thousand introns conserved between animals and A. thaliana or P. falciparum.

#### Author's response

*I included a comparison of the different method used and the results that were obtained in both mentioned articles. The method used in my article is quite stringent in the way that it requires high sequence similarity in a short region. The basic concept of an ancient intron, i.e. an intron at the same position within a similar sequence was used, so not only the position but also the splice site sequence had to match. I expect that a more BLAST like search with gap-alignment of homologous amino acids would yield more conserved introns. The scope of the first part of the article was not to get an exhaustive set, but to confirm that they exist and to study some characteristics*.

4) Many of the interpretations seem to me quite speculative and not supported by the data. For example, by looking at so many introns, it is expected that some matches will occur just by chance. Some simulations aimed to draw a threshold for this numbers would be welcomed.

#### Author's response

*As now more explicitly mentioned in the article, there is a potential bias in the search method, which was investigated and shown in *Fig. 5* and in *Fig. 6* which was quantified. The results supported the hypothesis of the Exon theory, and I have been careful not to suggest that they prove introns-early. See also discussion of dr. Roy's review*.

5) The analysis on the occurrence of conserved intron positions in ancient genes is presented in a confusing way. I strongly recommend the author to re-write this. Nevertheless, I have serious concerns about the analysis. First, it seems that de Roos call a gene ancient when there is some conservation in a window of 10 residues. This window size should be increased to at least the average size of a protein domain. Furthermore, it does not seem that the CDD database was used here.

#### Author's response

*I rewrote the analysis. I call an intron ancient when it is located in a conserved sequence, i.e. there is splice site similarity (6/10) as well a overall gene similarity as measured over an additional 20 (2 × 10) amino acids, overall a similarity of at least 12/30. In my approach this yielded a strong selection of conserved genes, and most matched sequences were a member of a conserved protein domain and shared a similar phase*.

#### Author's response

*There was no a priori assumption of gene relatedness and the CDD database was indeed not used to find the conserved set, but was used to confirm the ancientness of the set. As *Fig. 6* shows, when only an 8/10 splice site similarity was used, most sequences were conserved as shown by the sequence similarity further up and downstream. Lowering this to 6/10 but requiring an additional 6/20 (total 12/30) selected only conserved genes. Even with only a similar splice site (2 × 3 identical) and no further similarity, the sequences were in 42.1% genes shared with prokaryotes and 52.6% were member of a conserved domain (see text)*.

6) The interpretation derived from the analyses on the ancientness of the genes identified is kind of circular. Of course, if a condition was established in the early stages of the pipeline that a match would be considered a match only if involved kingdoms that split a long time ago, I would expect to have my dataset enriched with ancient proteins.

#### Author's response

*The article consists in a way of two parts. In the first, I tried to get a set of conserved introns by specifically looking for them. Since I query for similar intron positions between sequences that diverged in the eukaryotic tree, the dataset will be enriched for ancient proteins. I then looked whether this enrichment is due to the presence of an intron, or solely intrinsic to the method used. As can be seen in the controls of *Figs 5, 6, 7, *the presence of an intron makes a difference both in conservation of phase and overall sequence. I conclude that the enrichment of these sequences is due to the presence of the introns, and not a result of the method. In the Results as well as in the Discussion section, I made this more clear*.

### Reviewer's report 3

Gáspár Jékely, European Molecular Biology Laboratory, Heidelberg, Germany

This paper describes a new method to identified conserved intron positions in distantly related eukaryotic taxa. It is based on looking at short protein sequence stretches around a splice site and finding similar sequences having an intron at the corresponding position, rather then defining and aligning orthologs and then mapping intron positions onto the alignment. Using this method the author identifies 218 introns with the same phase at homologous sites that probably trace back to early eukaryote evolution. The method is novel and interesting. I have some comments on how to improve the presentation and analysis of the results.

1) One problem is that the phylogenetic context is not defined explicitly. It is not enough to refer to kingdoms, especially in the case of protist. If an intron is shared by animals, fungi (and in addition e.g. by choanoflagellate protists), it does not mean, that it was there in the eukaryotic common ancestor. The phyletic distribution of each identified intron shared by two or more kingdoms should be checked against a simplified but rooted eukaryote tree including the species under study (best would be to take a tree rooted between Unikonts and Bikonts). Only this way can the author reliably reconstruct conserved intron positions that have most likely been already present in the last eukaryotic common ancestor.

#### Author's response

*In line with the comments of the other reviewers, I have weakened the conclusions about the timing of the origin of introns based on the results presented here. Instead, the article is placed more as additional support for the Exon theory of genes, by the demonstration of conserved introns in ancient genes such as phosphatases*.

2) All the identified conserved introns should be shown in a word or excel sheet as supplemental information.

#### Author's response

*I now made the entire set available for download, including a set that still includes homologous genes (e.g Arac10 and Arac13, or gpc2 and GapC represent strongly homologous sequences*.

3) "Shared introns in ancient protein domains"

When the results of the CDD searches are presented, they should be presented along with a control, preferably as a table or graph. Great care should be taken when designing a control set (or sets). It should be selected based on the same criteria, including the phyletic distribution. It is quite tricky, given that the 'result set' is heterogeneous in this regard. The control should be of similar sample size as well, and given the heterogeneity and the small sample the best would be to sample a control many times independently (e.g. to have five control sets of ~ 200 sequences that show the same degree of sequence similarity across the same phyletic distance as the 'result set'). The selection for highly conserved sequences will obviously result in an enrichment of conserved domains. The design of the controls is crucial for the correct interpretation of the results, and it should be explained in detail in the text.

#### Author's response

*I have added paragraphs explaining the method and results in more detail. The sample size was not correctly given and the sample size for the control was not included, and this was changed. The results in *Figs. 3* and *4* do not have a direct control, they represent potential conserved introns and represent a different set used in *Figs 6* and *7. *It was investigated whether this was the result of a bias in the query and just represent a subset of conserved introns in conserved genes. The quantitative experiments in *Figs. 6* and *7* show this potential bias in a controlled situation, where the only difference between the sets is the presence of an intron. The positive correlation between presence of an intron and ancientness of the sequence is taken as support for the hypothesis that introns were indeed ancient*.

4) "Preferred occurrence of conserved introns in ancient genes"

In the analysis to identify prokaryotic hits, only sequences having "a number of identical amino acids residues of 6 (2 × 3) around a (virtual) splice site" were used. This means 19 sequences. The analysis should best be repeated for the whole set (218 sequences), with the appropriate control set (see above).

#### Author's response

*As can be seen in the graph of *Fig. 6, *there is a small window where are difference can be seen in ancientness of the genes. A lower similarity (2 or 4 identical residues) would mainly select false-positives, a higher number will select only conserved sequences in both sets. If the whole set is taken, then any effect will disappear in the noise. This represents one of the problems in analyzing intron data: given the enormous introns loss and possibly gain during evolution, together with possible intron sliding and the continuous protein diversification, it is difficult to discern an ancient signal in this noise*.

On page 2 the author writes: "The strongest prediction of an introns-early scenario is the presence of conserved introns between orthologous proteins that diverged early in the eukaryotic lineage." I don't think it is correct. If introns originated early during eukaryote evolution, but still later then prokaryotic genes, i.e. not as building blocks of the first genes to be assembled, one would also expect to find many conserved introns between eukaryotic orthologs.

#### Author's response

*I changed the introduction. Although expected in introns-early, conserved introns are compatible with introns-late. As now mentioned in the article in the last paragraph of the Discussion, I believe that the ancestral genome of prokaryotes and eukaryotes was a eukaryotic-like genome with characteristics such as introns and the RNA relics (e.g. the ribosome and the spliceosome). In that respect, early eukaryotic and ancient would be synonymous*.

Page 2/3: "It was found that conserved introns are frequently and specifically found in ancient protein domains" the word 'specifically' is too strong here, it would mean that conserved introns are only found in ancient domains, which is not the case (it is 53%).

#### Author's response

*Changed to 'positively correlated with sequence ancientness'*.

The word 'ancient' is often used as a synonym of 'conserved' but it is of course not the same thing. E.g. page 5 "Thus, based on these results, introns seem to be preferentially located into ancient genes, in line with an introns-early scenario" these are rather conserved, then necessarily 'ancient' genes.

#### Author's response

*Checked for inappropriate references to ancient and conserved*.

page 7: "In conclusion, the data presented here indicate a high occurrence of introns in ancient genes, followed by a massive loss of introns later in evolution. " High occurrence sounds too strong, a total of 218 introns were identified. Intron loss was not addressed in the paper, this would require mapping the distribution of conserved introns onto a phylogenetic tree.

#### Author's response

*I changed this. My interpretation of the data, with an Exon theory point-of-view, is that the ancient domains contained many introns since multiple were found in phosphatases for example *(Fig. 4). *The relatively low number of conserved introns found (251) indicate that either many were lost during evolution, or the sequence diverged and were not picked up by the current method. In the article there are two sets shown. The first consists of a set of conserved introns, based on the requirement that any 6 out of 10 residues should be identical, the second set consists of 5 separate queries in which the exact position of the identical residues was determined*.

Page 7: "These results support the idea of ancient introns and are difficult to reconcile with an origin of introns late in evolution, although an insertion directly after the eukaryote-prokaryote split cannot be excluded based on the current results." I would rather say that the results nicely support the ancestral presence of introns in the eukaryote common ancestor. This is interesting enough, and the whole paper and discussion would be much better if the author didn't try to argue too strongly for a model, that is not strongly supported by the data, but rather discuss what one can really conclude from the results.

#### Author's response

*I have changed the focus on the distinction between introns-late and introns-early (which was not well-supported by the data presented) towards a support for the Exon theory of genes. I made more explicit that, seen in the light of introns-late, these data would not exclude an introns-late scenario*.

## Supplementary Material

Additional File 1 Sets of conserved intron positions. This file contains the two sets of conserved introns in Excel format used in this article and a Word document containing a short description of field names. Click here for file

## References

[B1] Mattick JS (1994). Introns: evolution and function. Curr Opin Genet Dev.

[B2] Logsdon JM (1998). The recent origins of spliceosomal introns revisited. Curr Opin Genet Dev.

[B3] Rzhetsky A, Ayala FJ (1999). The enigma of intron origins. Cell Mol Life Sci.

[B4] Fedorova L, Fedorov A (2003). Introns in gene evolution. Genetica.

[B5] Doolittle WF (1987). Genes in pieces: were they ever together?. Nature.

[B6] Gilbert W (1987). The exon theory of genes. Cold Spring Harb Symp Quant Biol.

[B7] Rogers JH (1990). The role of introns in evolution. FEBS Lett.

[B8] Patthy L (1991). Exons–original building blocks of proteins?. Bioessays.

[B9] Palmer JD, Logsdon JM (1991). The recent origins of introns. Curr Opin Genet Dev.

[B10] Cavalier-Smith T (1991). Intron phylogeny: a new hypothesis. Trends Genet.

[B11] Cho G, Doolittle RF (1997). Intron distribution in ancient paralogs supports random insertion and not random loss. J Mol Evol.

[B12] De Souza SJ, Long M, Klein RJ, Roy S, Lin S, Gilbert W (1998). Toward a resolution of the introns early/late debate: only phase zero introns are correlated with the structure of ancient proteins. Proc Natl Acad Sci USA.

[B13] De Souza SJ (2003). The emergence of a synthetic theory of intron evolution. Genetica.

[B14] Roy SW (2003). Recent evidence for Exon Theory of Genes. Genetica.

[B15] Rodriguez-Trelles F, Tarrio R, Ayala FJ (2006). Origins and evolution of spliceosomal introns. Annu Rev Genet.

[B16] Reanney D (1979). RNA splicing and polynucleotide evolution. Nature.

[B17] Poole AM, Jeffares DC, Penny D (1998). The path from the RNA world. J Mol Evol.

[B18] De Roos ADG (2005). Origins of introns based on the definition of exon modules and their conserved interfaces. Bioinformatics.

[B19] Roy SW, Gilbert W (2006). The evolution of spliceosomal introns: patterns, puzzles and progress. Nat Rev Genet.

[B20] Kersanach R, Brinkmann H, Liaud MF, Zhang DX, Martin W, Cerff R (1994). Five identical intron positions in ancient duplicated genes of eubacterial origin. Nature.

[B21] Fedorov A, Cao X, Saxonov S, de Souza SJ, Roy SW, Gilbert W (2001). Intron distribution difference for 276 ancient and 131 modern genes suggests the existence of ancient introns. Proc Natl Acad Sci USA.

[B22] Fedorov A, Merican AF, Gilbert W (2002). Large-scale comparison of intron positions among animal, plant, and fungal genes. Proc Natl Acad Sci USA.

[B23] Rogozin IB, Wolf YI, Sorokin AV, Mirkin BG, Koonin EV (2003). Remarkable interkingdom conservation of intron positions and massive, lineage-specific intron loss and gain in eukaryotic evolution. Curr Biol.

[B24] Coghlan A, Wolfe KH (2004). Origins of recently gained introns in Caenorhabditis. Proc Natl Acad Sci USA.

[B25] Babenko VN, Rogozin IB, Mekhedov SL, Koonin EV (2004). Prevalence of intron gain over intron loss in the evolution of paralogous gene families. Nucleic Acids Res.

[B26] Roy SW, Penny D (2006). Smoke without fire: most reported cases of intron gain in nematodes instead reflect intron losses. Mol Biol Evol.

[B27] Jeffares DC, Mourier T, Penny D (2006). The biology of intron gain and loss. Trends Genet.

[B28] Koonin EV (2006). The origin of introns and their role in eukaryogenesis: a compromise solution to the introns-early versus introns-late debate?. Biol Direct.

[B29] Blake CC (1979). Exons encode protein functional units. Nature.

[B30] Darnell JE, Doolittle WF (1986). Speculations on the early course of evolution. Proc Natl Acad Sci USA.

[B31] Gilbert W, Marchionni M, McKnight G (1986). On the antiquity of introns. Cell.

[B32] Dorit RL, Schoenbach L, Gilbert W (1990). How big is the universe of exons?. Science.

[B33] Long M, Rosenberg C, Gilbert W (1995). Intron phase correlations and the evolution of the intron/exon structure of genes. Proc Natl Acad Sci USA.

[B34] Go M (1985). Protein structures and split genes. Adv Biophys.

[B35] De Souza SJ, Long M, Schoenbach L, Roy SW, Gilbert W (1996). Intron positions correlate with module boundaries in ancient proteins. Proc Natl Acad Sci USA.

[B36] Roy SW, Nosaka M, de Souza SJ, Gilbert W (1999). Centripetal modules and ancient introns. Gene.

[B37] Sakharkar M, Passetti F, de Souza JE, Long M, de Souza SJ (2002). ExInt: an Exon Intron Database. Nucleic Acids Res.

[B38] Roy SW, Gilbert W (2005). Resolution of a deep animal divergence by the pattern of intron conservation. Proc Natl Acad Sci USA.

[B39] Sakharkar MK, Chow VT, Kangueane P Distributions of exons and introns in the human genome. In Silico Biol.

[B40] Endo T, Fedorov A, de Souza SJ, Gilbert W (2002). Do introns favor or avoid regions of amino acid conservation?. Mol Biol Evol.

[B41] Nguyen HD, Yoshihama M, Kenmochi N (2006). Phase distribution of spliceosomal introns: implications for intron origin. BMC Evol Biol.

[B42] Roy SW, Gilbert W (2005). Complex early genes. Proc Natl Acad Sci USA.

[B43] Slamovits CH, Keeling PJ (2006). A high density of ancient spliceosomal introns in oxymonad excavates. BMC Evol Biol.

[B44] Archibald JM, O'Kelly CJ, Doolittle WF (2002). The chaperonin genes of jakobid and jakobid-like flagellates: implications for eukaryotic evolution. Mol Biol Evol.

[B45] Gilbert W, Glynias M (1993). On the ancient nature of introns. Gene.

[B46] De Roos ADG (2006). The origin of the eukaryotic cell based on conservation of existing interfaces. Artif Life.

[B47] Poole A, Jeffares D, Penny D (1999). Early evolution: prokaryotes, the new kids on the block. Bioessays.

[B48] Marchler-Bauer A, Anderson JB, DeWeese-Scott C, Fedorova ND, Geer LY, He S, Hurwitz DI, Jackson JD, Jacobs AR, Lanczycki CJ, Liebert CA, Liu C, Madej T, Marchler GH, Mazumder R, Nikolskaya AN, Panchenko AR, Rao BS, Shoemaker BA, Simonyan V, Song JS, Thiessen PA, Vasudevan S, Wang Y, Yamashita RA, Yin JJ, Bryant SH (2003). CDD: a curated Entrez database of conserved domain alignments. Nucleic Acids Res.

[B49] Dibb NJ, Newman AJ (1989). Evidence that introns arose at proto-splice sites. EMBO J.

