# Supplementary material for: Conserved intron positions in ancient protein modules
Source: Biol Direct. 2007 Feb 8;2:7. doi: 10.1186/1745-6150-2-7 (PMC1800838; doi:10.1186/1745-6150-2-7)
Supplement: Additional File 1 — Sets of conserved intron positions. This file contains the two sets of conserved introns in Excel format used in this article and a Word document containing a short description of field names. [file 1745-6150-2-7-S1.zip › Conserved sets/conserved set table fields explanation.doc]

# Table cset

Complete set of genes with conserved splice sites. Shows the homologous gene pairs from sequences with conserved introns, used in Fig. 2 and 3.

| Axn_1 | GenBank accession number #1 |
| --- | --- |
| Axn_2 | GenBank accession number #2 |
| Species_1 | species |
| Species_2 | species |
| Taxon_id_1: | used to refer to taxon name in reference table |
| Taxon_id_2 | used to refer to taxon name in reference table |
| Left3_1 | last 3 residues before the virtual splice site; used to sort the data |
| Right3_1 | first 3 residues after the virtual splice site; used to sort the data |
| Aaleft_1 | last 15 residues before the virtual splice site |
| Aaright_1 | first 15 residues after the virtual splice site |
| Aaleft_2 | last 15 residues before the virtual splice site |
| Aaright_2 | first 15 residues after the virtual splice site |
| Phase_1 | Phase intron |
| Phase_2 | Phase intron |
| Seq-annot_1 | sequence annotation |
| Seq_annot_2 | sequence annotation |
| Exon1_1 | Exon number before the splice site gene #1 |
| Exon2_1 | Exon number after the splice site gene #1 |
| Exon1_2 | Exon number before the splice site gene #2 |
| Exon2_2 | Exon number after the splice site gene #2 |
| Exon1size_1 | Idem exon size |
| Exon2size_1 | Idem exon size |
| Exon1size_1 | Idem exon size |
| Exon2size_2 | Idem exon size |
| Homol_left_1 | number of homologous residues in left ‘general homology’ region, cf. Fig. 2 |
| Homol_right_2 | number of homologous residues in right ‘general homology’ region, cf. Fig. 2 |
| Non-Rednt | Identifies non-reduntant taxon combination, excluded from analysis |
